# A Simple Graphene NH_3_ Gas Sensor via Laser Direct Writing

**DOI:** 10.3390/s18124405

**Published:** 2018-12-13

**Authors:** Dezhi Wu, Qianqian Peng, Shan Wu, Guangshun Wang, Lei Deng, Huiling Tai, Lingyun Wang, Yajie Yang, Linxi Dong, Yang Zhao, Jinbao Zhao, Daoheng Sun, Liwei Lin

**Affiliations:** 1 Department of Mechanical and Electrical Engineering, Xiamen University, Xiamen 361005, China; qianp77@stu.xmu.edu.cn (Q.P.); 34520162201412@stu.xmu.edu.cn (S.W.); wanggs@stu.xmu.edu.cn (G.W.); 32020151153660@stu.xmu.edu.cn (L.D.); wangly@xmu.edu.cn (L.W.); sundh@xmu.edu.cn (D.S.); lwlin@berkeley.edu (L.L.); 2 State Key Laboratory of Electronic Thin Films and Integrated Devices, University of Electronic Science and Technology of China, Chengdu 610054, China; taitai1980@uestc.edu.cn (H.T.); yangyajie@uestc.edu.cn (Y.Y.); 3 Key Laboratory of RF Circuits and System of Ministry of Education, Hangzhou Dianzi University, Hangzhou 310018, China; donglinxi@hdu.edu.cn; 4 Department of Chemical Engineering, Xiamen University, Xiamen 361005, China; jbzhao@xmu.edu.cn; 5 Department of Mechanical Engineering, University of California at Berkeley, CA 94703, USA

**Keywords:** ammonia gas sensor, graphene, laser direct writing

## Abstract

Ammonia gas sensors are very essential in many industries and everyday life. However, their complicated fabrication process, severe environmental fabrication requirements and desorption of residual ammonia molecules result in high cost and hinder their market acceptance. Here, laser direct writing is used to fabricate three parallel porous 3D graphene lines on a polyimide (PI) tape to simply construct an ammonia gas sensor. The middle one works as an ammonia sensing element and the other two on both sides work as heaters to improve the desorption performance of the sensing element to ammonia gas molecules. The graphene lines were characterized by scanning electron microscopy and Raman spectroscopy. The response and recovery time of the sensor without heating are 214 s and 222 s with a sensitivity of 0.087% ppm^−1^ for sensing 75 ppm ammonia gas, respectively. The experimental results prove that under the optimized heating temperature of about 70 °C the heaters successfully help implement complete desorption of residual NH_3_ showing a good sensitivity and cyclic stability.

## 1. Introduction

Ammonia gas plays a vital role in numerous fields, including nitric acid manufacturing, petrochemical industries, plastics, explosives, textiles, as a refrigerating fluid and in gallium nitride, etc [1,2], but exposure to it at higher concentrations will lead to serious damage to the human eyes, skin and respiratory system [3]. The concentration limit for exposure to ammonia for a long time (8 h) is 25 ppm and the limit for short-term (15 min) exposure is 35 ppm. Therefore, it is important to detect ammonia gas when needed in applications to guarantee our safety.

In the past several decades, many researchers have endeavored to develop numerous differerent NH_3_ sensors with different features such as materials, structures and fabrication methods and great advances have been achieved [4]. Various conductive polymers (polyaniline (PANI), polypyrrole, poly(*m*-aminobenzene sulfonic acid) (PABS), poly(3-hexylthiophene)) [5,6,7], inorganic (TiO_2_, SnO_2_, ZnO, V_2_O_5_, In_2_O_3_, WO_3_, CeO_2_, Carbon nanotubes, graphene) [8,9,10,11,12,13] or hybrid composite materials (PANI/MWCNT, PANI/SnO_2_, PANI/ZnO, PABS/SWCNT) with synergistic effects [14,15,16,17] have been verified to be capable of sensing ammonia gas. Conducting polymers have attracted a lot of attention due to their unique electrical properties. The relative ease of synthesis and room temperature operation makes conducting polymers promising candidates in the field of gas sensing [18,19]. However, their long recovery time and incomplete desorption of ammonia gas molecules limit their application [20]. The fabrication of conductive polymers/metal oxide composites—as a potential solution to overcome the shortcomings of conductive polymers, has been widely studied for gas sensing applications. Hybridization with metal oxides greatly enhances the gas sensor performance of conductive polymers [21]. The metal oxide-based sensors are commercially available for various applications, but suffer from a problem of high operational temperature limiting long-term device stability. At low temperatures, the response recovery time of the sensor typically slows down. In addition to these, carbon nanostructures, including CNTs and graphene, are other potential candidates for ammonia gas sensing because of their structure and large surface area. Nevertheless, the path of these devices towards commercialization is hindered by the complexity and cost of their fabrication process. For most sensors, chemicals that pollute our environment are used, and the fabrication processes such as coating [22], synthesis [5], printing [23], chemical vapor deposition [24] and electrospinning [25] etc. are complicated. It is worth noting that gas molecules cannot be completely desorbed when sensors recover at room temperature, resulting in poor stability and long recovery times, which are common problems of gas sensors [20]. Several methods have been extensively explored to improve the desorption of the residual molecules. For example, Li et al. utilized ultraviolet light exposure, which reduced the energy barrier of desorption, to accelerate the recovery [26]. Gautam et al. promoted the desorption behavior of ammonia from a graphene surface by illuminating the graphene-based NH_3_ sensor with an infrared source. The effect was attributed to the absorption of infrared light by graphene to generate a charge carrier [27]. Most other reports also accelerated the desorption process with additional heating, which is based on the principle of heat exciting gas molecules [28]. While these auxiliary devices can be used to effectively achieve complete desorption in shorter time, unfortunately they make the sensor bulky and complex, so sensor-to-heater integration is undoubtedly a much more elegant approach.

Graphene, a two-dimensional material, has outstanding potential as a gas sensor [29]. Graphene-based ammonia gas sensors prepared by various methods such as chemical vapor deposition (CVD) [30], mechanical exfoliation of graphite [31] and reduction of graphene oxide [32] have been reported. Due to its ultra-high surface area, the electronic properties show strong dependence on surface absorbents including gas molecules, which can alter the carrier density of graphene. When gas molecules are adsorbed on the surface of graphene, the graphene sheet can act as an excellent electron acceptor or donor (for example, NO_2_ acts as an electron acceptor and NH_3_ acts as an electron donor on graphene), which leads to the transfer of electrons. The transfer between graphene and adsorbed gas molecules changes the resistance and carrier density of graphene. According to this principle, graphene is considered to be useful for detecting a variety of molecules. However, its low productivity, severe manufacturing requirements and possible transfer process result in costly barriers to its commercial application. Recent literatures reported that graphene-based materials can also can be made from commercial polyimide (PI) film by laser direct writing [33], making them potentially cost-effective for high-performance charge storage devices [34] and sound-sensing [35]. Here we propose a facile method to fabricate three parallel porous 3D graphene lines on a thin PI tape by using the laser direct writing technique to construct a flexible ammonia gas sensor, in which the sandwiched line acts as a sensing element, and the other two lying beside the sensing element act as heaters to help achieve complete desorption of ammonia molecules. Such a structure all-graphene scheme can be fabricated in a one-step process and free of additional assemblies. The graphene lines induced via laser direct writing were characterized by means of Scanning Electron Microscopy (SEM) and Raman spectroscopy. The sensing performance of the sensor including response/recovery time, sensitivity and stability were evaluated without an applied voltage to heaters under different ammonia concentration ranging from 75 to 400 ppm at first. The resistance could not revert to the original value illustrating that there are residual NH_3_ molecules on the sensing element. Then after being heated at an optimized temperature of 70 °C, the absorption and desorption behaviors of the sensor are investigated and the results shows that it exhibits complete desorption of ammonia molecules and good stability.

## 2. Experiments

### 2.1. Gas Sensor Fabrication

**Figure 1:**
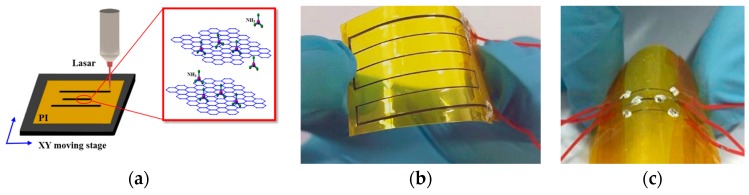
(**a**) A diagram of laser direct writing; (**b**) A photo of a flexible zigzag pattern fabricated by laser direct writing; (**c**) A photo of a test sample of the sensor.

As illustrated in Figure 1a, the experimental laser direct-writing process setup consists of a CO_2_ laser system with wavelength of 10.6 μm (J48-2W, Synrad, Marcotio, Washington, USA), an XY moving stage and a target substrate. The focused beam spot size is reduced to about 0.1 mm when the focal distance is set to be 5 cm by adjusting the mirror s and focus lens. PI film tape (Hangzhou Surmount Science & Technology, Hangzhou, China) was attached to the target substrate on the XY moving stage. When the focused thin laser beam was focussed on the PI film with help of the moving stage, converting PI into graphene with the designed patterns. The principle is that a laser could produce extremely high local temperatures of more than 2500 °C and high local pressure, which facilitates carbonization and graphitization of the polyimide surface. Under ambient conditions, the oxygen and water molecules around the direct writing area will react with graphitic carbon to release some CO and CO_2_ gases and then porous structures are formed. Furthermore, the heterogeneous distribution of oxygen, moistures and unpredictable rapid heat flow result in a complicate 3D porous morphology [33]. 

According to our previous work [36], we have explored the effect of the key processing parameters on the properties of the formed graphene and the optimized laser power density, whereby the moving speed and pulse width were set to be 7.27 kw/cm^2^, 1.6 mm/s and 357 μs respectively. A laser irradiated zigzag pattern is illustrated in Figure 1b as an example and the black line is graphene. A gas sensor consisting of three parallel lines with a pitch of 2 mm was fabricated, in which the lengths of the middle and the other two lines were 5 mm and 1 cm, respectively, as depicted in Figure 1c. Conductive silver paste was coated at both ends of the graphene lines.

### 2.2. Characterization Techniques

The patterned lines were firstly characterized by SEM. Raman spectra was recorded by a CRM200 Raman System (WITEC, Ulm, Germany) and the excitation wavelength is 532 nm with spot size of ∼ 500 nm and 2.5 mw at the source. Their thermal effect on the sensor was characterized by an infrared thermal imager (Fotric-226, FLIR, Wilsonville, Oregon, USA).

### 2.3. Tests of the Gas Sensor

**Figure 2:**
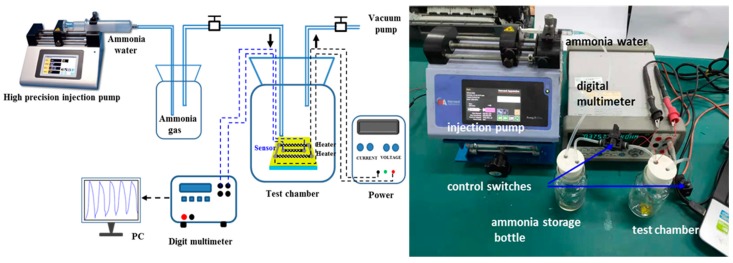
A schematic diagram and a photo of the NH_3_ gas sensor testing setup.

As illustrated in Figure 2, our homemade gas sensor test system, described elsewhere [37,38,39], mainly consists of a high precision injection pump (Harvard-11 Pico Plus, Horrison, Massachusetts, USA), a bottle for ammonia storage, a test chamber, control switches, a vacuum pump, a digital multi-meter and a computer. Ammonia water is contained in a syringe. The graphene-based ammonia gas sensor was sealed in the test chamber at ambient atmosphere and room temperature. The pump is used to pump different volumes of ammonia water into the ammonia storage bottle to produce gas vapors with different concentrations for testing. The first control switch is then turned on and gas vapor is passed into the test chamber. The volume of ammonia gas produced can be calculated by the volume and purity of the pumped ammonia, and the ratio of the volume of ammonia gas to the total volume of the storage bottle and the test chamber is the concentration of ammonia. The other switch controls the connectivity with the outside air or the air pump to clean the ammonia in the system after a single test. The digital multimeter (Agilent 34410A, Santa Rosa, California, USA) can monitor the resistance of the sensor when it was exposed to alternate cycles of NH_3_ (test gas) and atmosphere across the two terminals.

## 3. Results and Discussions

### 3.1. Structure Characterization

**Figure 3:**
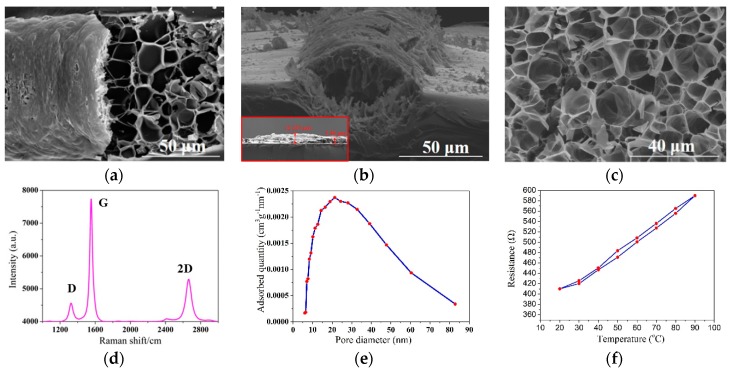
(**a**) SEM image of the laser irradiated PI; (**b**) Side view of (**a**); (**c**) Amplified view of (**a**); (**d**) Raman spectroscopy of the laser irradiated PI; (**e**) The pore diameter distribution curve of the laser direct-write PI; (**f**) Resistance change of the laser irradiated PI with temperature.

The morphologies and properties of the laser direct-write PI were characterized by SEM and Raman spectroscopy. From Figure 3a,b, it can be seen that the irradiated PI looks like a semi-cylinder with a coarse outer surface and its width is estimated to be about 120 μm. The maximum height of graphene observed from the surface of the PI substrate is about 12 μm as shown in the inset of Figure 3b. Figure 3c exhibits the inner surface of the sample which looks like honeycombs with porous structures resulting from the rapid liberation of gaseous products. The Raman spectra shown in Figure 3d presents three characteristic peaks, a D peak at ~1346 cm^−1^, a G peak at ~1581 cm^−1^, and a 2D peak at ~2663 cm^−1^. The positions of these three characteristic peaks are substantially consistent with that of graphene [40]. The D peak reflects the defects inside the graphene, and the G peak is related to the crystallinity and order of the graphene, and the 2D peak corresponds to the degree of graphene. As depicted in Figure 3e, the pore size distribution of the laser direct-write PI ranges from 6 to 84 nm, and their diameters mainly concentrate at about 21 nm. According to BET formula, its specific surface area is calculated to be approximately 16.83 m^2^/g, which is slightly larger than that of the coated polyaniline gas sensor previously reported [41]. In addition, the electrical resistance change of the laser-induced graphene element (length 20 mm) with temperature is described in Figure 3f. It can be found that the initial resistance of the graphene element is approximately 410 Ω and the resistance rises to about 590 Ω when heated up to 90 °C. While the temperature dropped, the resistance was slightly higher than that in heating process.

### 3.2. Response/Recovery Behavior and Its Sensitivity without Heating

Electron transport in graphene is very sensitive to NH_3_ molecules because its ultra large surface area will bring more opportunities for ammonia molecules, acting as donators, to attach to graphene surface [42,43]. A good example is that Schedin et al. experimentally proved that monolayer graphene can detect gaseous species to one single molecular level [31]. When ammonia molecules are absorbed by graphene, the NH_3_ molecules provide electrons to compound with the hole in the graphene conduction belt resulting in electronic resistance increase of the sensing element. The laser direct-written graphene lines provide numerous 3D porous micro/nano structures, which facilitate adsorption of ammonia gas molecules and their sensitivity will become higher.

The sensing response is defined as ΔR/R, where $\Delta R=R_{g}-R$, $R_{g}$ and R represent the real-time resistance to the ammonia gas and its initial electrical resistance to atmosphere respectively. The definition of the gas sensitivity is defined as:

(1) $$S=\frac{\Delta R}{R\times \Delta C},$$

where ΔC is the concentration difference of ammonia gas.

**Figure 4:**
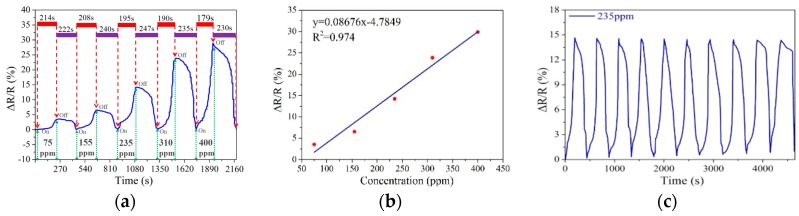
(**a**) The real-time response/recovery behavior of the sensor to various concentrations of ammonia gas from 75 to 400 ppm; (**b**) The normalized resistance changes of the sensor to various concentrations of ammonia gas from 75 to 400 ppm; (**c**) Real-time cycling response of the sensor with 120 μm width to 235 ppm NH_3_ gas.

In order to evaluate the function of the two heaters, the voltage applied on the heaters was set to be zero at first to study the performance of the sensor alone. Figure 4a presents the real-time response/recovery behavior of the graphene sensors to ammonia gas with various concentrations from 75 to 400 ppm. When the concentration was 75, 155, 235, 310 and 400 ppm, the normalized real-time response of the sensor increased to 3.552%, 6.53%, 14.22%, 23.85% and 29.87%, and the response/recovery time were 214/222 s, 208/240 s, 195/247 s, 190/235 s and 179/230 s, respectively. From Figure 4b, a good linear relationship between the resistance response and NH_3_ concentration could be observed, and the sensitivity of the sensor was about 0.087% ppm^−1^. Therefore it can be concluded that the change rate of the electrical resistance has a positive linear relationship with the concentration of ammonia gas, but the relationship between response/recovery time and gas concentration is on the contrary.

Subsequently, real-time cycling response test of the sensor was performed in ten cycles of periodic presence of NH_3_ gas with concentration about 235 ppm. Figure 4c shows that the maximum of the normalized resistance change remained at about 15%, but there still were two visible changes. Firstly, the recovery time of the sensor increased gradually from 246 s to 269 s when the sensor started to sense gas initially at the 10th cycle. Secondly, the resistance cannot return to its initial value and it showed drifting that became larger with increasing cycle times, indicating that the residual ammonia molecules in the sensing element did not desorb completely, so it is necessary to apply a voltage to the heaters to increase the temperature to promote desorption and improve its cycling stability.

### 3.3. Ammonia Molecules Desorption of the Sensor with Heating

**Figure 5:**
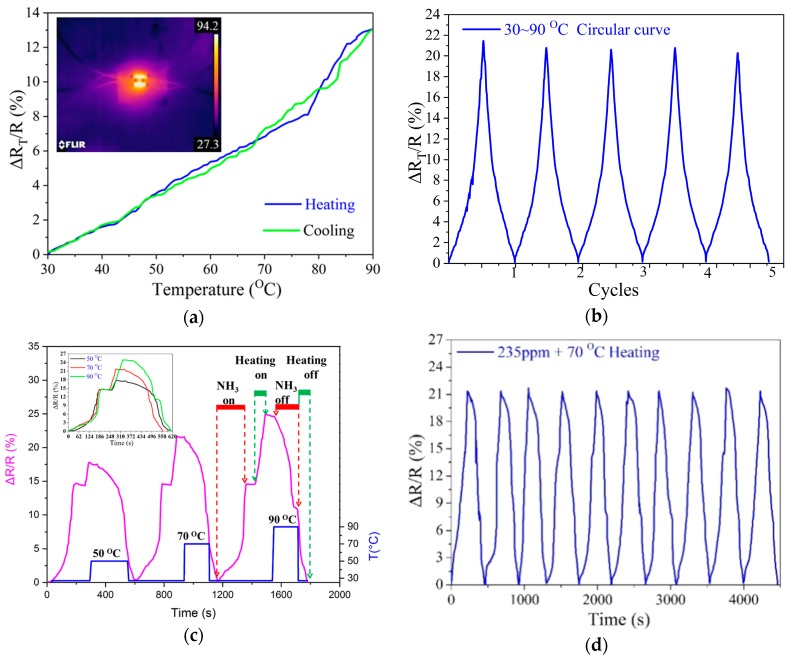
(**a**) The normalized resistance change of the graphene gas sensing element caused by thermal effect (heating and cooling); (**b**) Cycling response of the sensor to 235 ppm NH_3_ gas during heating and cooling; (**c**) The normalized real-time resistance response/recovery behavior of the sensor to 235 ppm NH_3_ at various desorption temperature from 50 to 90 °C; (**d**) Real-time cycling response of the sensor to 235 ppm NH_3_ gas at 70 °C.

The effect of heating temperature on the electronic resistance of the gas sensing element was investigated at first by performing heating–cooling cyclic tests within 30–90–30 °C. Figure 5a shows the heating–cooling process has an effect on the resistance. During the heating process, a 15 V DC voltage was applied to the two heaters, and during cooling process, the voltage potential was turned off to cool naturally. The resistance difference is defined as ΔR_T_. The normalized resistance difference changes ΔR_T_/R with temperature are nearly linear. When the thermal radiation temperature was 90 °C, the ΔR_T_/R of gas sensing element was about 13.07%. This may be due to the change in the intercalation of graphene by thermal instability, which in turn changes its resistance. The inset of Figure 5a is a typical thermal infrared image of temperature distribution at a center temperature of 70 °C. It can be seen that the thermal radiation makes the temperature around the entire even distributed. Figure 5b shows the reversibility of the heating–cooling profile in the temperature range of 30~90~30 °C for the gas sensing element, indicating that the gas sensing element has good temperature cycling performance.

In order to confirm an appropriate heating temperature around the sensing element and improve heating efficiency, we investigated the heating temperatures on response performance of the sensor by changing the temperature to be 50, 70 and 90 °C under an ammonia gas concentration about 235 ppm. A series of voltages of 3.5V, 4.7V, and 6.2V was applied to the heaters to maintain the temperature around the middle graphene gas sensor at 50 °C, 70 °C, and 90 °C, respectively. Firstly, the resistance of the sensing element increased and kept at about 15% increment when the graphene sensor was exposed to 235 ppm NH_3_. Secondly, the resistance further rose to another stable value due to the thermal effect when the sensor was heated to the set temperature by applying a DC voltage onto the heaters. Thirdly, the ammonia gas in the test chamber was pumped outside and we can see that the resistance decreased gradually to a fixed value because NH_3_ was desorbed at the set temperature. Finally, the power is turned off and the resistance further decreased to its initial value state as the temperature retrieved to room temperature. The inset image is the horizontal contrast of Figure 5c, which shows the desorption capacity at the central temperature of 50, 70, 90 °C, respectively. At 50 °C, the final resistance change rate of the graphene gas sensor element is about 0.400%, indicating that the gas sensor element is not able to completely desorb the ammonia gas. At 70 °C and 90 °C, the final resistance change rate is nearly zero, and the resistance returned to its initial value, which means that the gas sensing element can completely desorb the ammonia gas. However, since the process of raising the temperature to 90 °C and cooling down takes a long time, which affects the detection time. Therefore, the overall detection time is shorter at 70 °C. Figure 5d shows the reversibility of the sensor over 10 cycles with 70 °C in 235 ppm of NH_3_ during desorption and the resistance can recover to its initial value. It can be concluded that the sensor demonstrated good resolution, excellent sensitivity, good thermal stability and appreciable response/recovery time because the graphene heaters helped to implement complete desorption of ammonia gas when the temperature is more than 70 °C.

## 4. Conclusions

A CO_2_ laser beam was utilized to scribe PI tape to successfully fabricate three parallel graphene lines for an ammonia gas sensor, in which the middle one acted as a sensing element and the other two acted as heaters to implement complete desorption of the ammonia molecules absorbed on graphene surface. SEM and Raman spectra proved that the irradiated micropatterns were changed into graphene with 3D porous structures. Without a heating process, its response/recovery time were 214/222 s, respectively, with the sensitivity of ca. 0.087% ppm^−1^. With the optimized 70 °C heating temperature around the sensing element, residual ammonia molecules were completely detached from the graphene surface, and the sensitivity and cyclic stability were greatly improved. Thus, such method might be a possible candidate to fabricate graphene-based ammonia gas sensors, which will be widely used in future applications including environmental detectors and wearable nose-sensors.
